# Cellular senescence impact on immune cell fate and function

**DOI:** 10.1111/acel.12455

**Published:** 2016-02-22

**Authors:** Rita Vicente, Anne‐Laure Mausset‐Bonnefont, Christian Jorgensen, Pascale Louis‐Plence, Jean‐Marc Brondello

**Affiliations:** ^1^ INSERM, U1183, IRMBMontpellier CedexFrance; ^2^ University of MontpellierMontpellierFrance; ^3^ CHRU de Montpellier, IRMBMontpellier CedexFrance

**Keywords:** cellular senescence, chronic and autoimmune diseases, immune cells, tissue homeostasis

## Abstract

Cellular senescence occurs not only in cultured fibroblasts, but also in undifferentiated and specialized cells from various tissues of all ages, *in vitro* and *in vivo*. Here, we review recent findings on the role of cellular senescence in immune cell fate decisions in macrophage polarization, natural killer cell phenotype, and following T‐lymphocyte activation. We also introduce the involvement of the onset of cellular senescence in some immune responses including T‐helper lymphocyte‐dependent tissue homeostatic functions and T‐regulatory cell‐dependent suppressive mechanisms. Altogether, these data propose that cellular senescence plays a wide‐reaching role as a homeostatic orchestrator.

## Introduction

In the 1960s, Hayflick and Moorhead first used the term ‘cellular senescence’ to describe the limited replicative potential of primary human fibroblasts *in vitro* (Hayflick & Moorhead, 1961). This phenotype was dependent on the telomere length and on the induction of two major cell cycle inhibitory pathways: the ATM/p53/p21^Waf1^ and the p16^INK4a^/pRB signaling cascades. Fifty years later, we now know that cellular senescence occurs in response to a large variety of extrinsic and intrinsic signals that can be associated or not with telomere erosion and the presence of permanent DNA damage (for review, see Muñoz‐Espín & Serrano, 2014; Salama *et al*., 2014). Senescent cells are detected during embryo tissue development, in highly differentiated cell types as well as during tissue repair, tumor suppression responses, and age‐associated loss of tissue functions (for review, see Muñoz‐Espín & Serrano, 2014; Salama *et al*., 2014). The senescence‐promoting signaling cascades lead to three features found in all senescent cells: (i) permanent cell cycle arrest, associated with (ii) chromatin alterations, leading to the (iii) establishment of a specific secretome, called senescence‐associated secretory phenotype (SASP). Through the production of specific paracrine and autocrine factors, such as chemokines (CCL2/MCP‐1, TNF‐α, IFN‐γ GRO‐α, IL‐8, IL‐6), growth and differentiation factors (TGF‐β and HGF), and matrix‐remodeling enzymes (MMP1/3/10/13), this SASP allows the cross‐talk between senescent cells and neighboring cells. The SASP also triggers the detection, elimination, and rapid replacement of senescent cells by the homeostatic protective system of each tissue. Altogether, these recent findings offer new insights into the complex roles of cellular senescence during ontogenesis as well as in physiological and pathological situations conserved in many species (Campisi & Robert, 2014; Muñoz‐Espín & Serrano, 2014; Salama *et al*., 2014).

Cellular senescence, especially telomere‐dependent senescence, has been known for years to occur in immune cells (for review, Chou & Effros, 2013). Recent work suggests that the onset of cellular senescence can also be used by the immune system to guide immune cell fate decision, regulate immune responses, and control tissue homeostasis during the entire life of an individual. We review here these new findings that help us to better understand the role(s) of cellular senescence in tissue homeostasis and in chronic inflammatory disorders.

## Cellular senescence and immune cell fate decision

Cellular senescence was originally described as an irreversible cell cycle arrest even in the presence of growth factor stimulation (Hayflick & Moorhead, 1961). This property makes senescent cells clearly different from quiescent cells (Campisi & Robert, 2014). One gene locus, namely *CDKN2A,* which encodes for two unrelated proteins p16^INK4a^ and p14/p19^ARF^, is involved in this senescence‐induced cell cycle arrest (Krishnamurthy *et al*., 2004). Indeed, by inhibiting cyclin‐dependent kinases (CDKs), p16^INK4a^ maintains the retinoblastoma family members (pRB, p107, and p130) in their transcriptionally repressive forms, thus preventing G1/S cell cycle progression (Gil & Peters, 2006). On the other hand, by interfering with MDM2 (mouse double minute 2)‐dependent degradation of p53, p14/p19^ARF^ controls p53/p21^WAF1^‐induced G1 or G2 cell cycle arrest (Gil & Peters, 2006). Remarkably, using knockout mice models for this locus, it was possible to reveal that these senescence features could be associated with the terminal differentiation program in some specific cell types including keratinocytes (Paramio *et al*., 2001; Bachoo *et al*., 2002), chondrocytes (Philipot *et al*., 2014), myofibers (Pajcini *et al*., 2010), and also megakaryocytic cells (Muñoz‐Espín & Serrano, 2014). Megakaryocytic cells, myeloid‐derived immune cells from which blood platelets originate, are required for wound healing and immune responses (Besancenot *et al*., 2010), opening new avenues to explore features of senescence in other types of immune cells.

### Cellular senescence onset and macrophages polarization

Macrophages are key players in the defense mechanisms against pathogens and play a central role in inflammation and host defense. They also fulfill homeostatic functions including tissue remodeling and the resolution of tissue damage. Heterogeneity of the macrophage lineage has long been recognized (Gordon & Taylor, 2005), and they have different functional roles depending on their tissue location and the inflammatory environment that drives their activation (Davies *et al*., 2013). In this context, macrophage activation has been conventionally categorized into pro‐inflammatory M1 macrophages, induced by IFN‐γ and toll‐like receptor (TLR) ligands, and the alternatively activated anti‐inflammatory M2 macrophages, induced by IL‐4/IL‐13 (Biswas & Mantovani, 2010; Gordon & Martinez, 2010). M1 are efficient producers of toxic effector molecules, pro‐inflammatory cytokines, and chemokines that kill pathogens and virus‐infected cells as well as senescent or cancer cells (Hoenicke & Zender, 2012; Davies *et al*., 2013). In contrast, M2 cells have high levels of scavenger receptors and play a role in allergy, parasite clearance, dampening of inflammation, tissue remodeling, angiogenesis, immunoregulation, and tumor progression (Mantovani *et al*., 2013; Wynn *et al*., 2013). Although a detailed description of these categories is beyond the scope of this review, it is clear that this description is overly simplistic and that macrophages are very plastic cells of the hematopoietic system and exist in a spectrum of states that depend on their tissue microenvironment (Mosser & Edwards, 2008).

Interestingly, the expression and role of senescence markers, such as p16^INK4a^ and p14/p19^ARF^, in murine bone marrow‐derived macrophages (BMDM), as well as in human adipose tissue macrophages, have been recently described (Cudejko *et al*., 2011; Fuentes *et al*., 2011). Cudejko *et al*. reported that p16^INK4a^ deficiency influences macrophage polarization *in vitro* and *in vivo* using chimeric mice infected by a parasite. Indeed, transcriptome analysis of p16^INK4a^‐deficient BMDMs revealed a dramatic down‐regulation of genes associated with inflammatory M1, such as IL‐6, and up‐regulation of genes associated with the M2 phenotype, such as ARG1 (arginase‐1), and Ym1/2 compared with wild‐type BMDM. Interestingly, incubation with IL‐4, the M2 polarization factor, further increased the expression of M2‐associated genes in p16^INK4a^‐deficient BMDM. Conversely, incubation with the classical M1 polarization factors, IFN‐γ and LPS, led to a decrease in IL‐6, TNF‐α, and MCP‐1 expression in p16^INK4a^‐deficient BMDM (Cudejko *et al*., 2011; Fuentes *et al*., 2011). Similarly, p16^INK4a^ expression was low in human adipose tissue macrophages and silencing p16^INK4a^ expression in monocyte‐derived macrophages increased mRNA expression of M2 markers such as *MRC1* and *AMAC1* (Fuentes *et al*., 2011). Moreover, in agreement with the murine studies, *in vitro* IL‐4‐polarized human M2 macrophages expressed lower levels of p16^INK4a^ than *in vitro* IFN‐γ‐polarized M1 (Cudejko *et al*., 2011; Fuentes *et al*., 2011). Similar polarizing properties were obtained with primary macrophages that can become senescent after 2 weeks of *in vitro* expansion, or upon ectopic p16^INK4a^ expression. Indeed, Murakami *et al*. (2012) recently showed that p16^INK4a^ expression suppresses LPS‐induced production of IL‐6 in mouse and human macrophages.

Altogether, these recent data point to cell autonomous roles of p16^INK4a^‐induced cellular senescence in macrophage polarization toward the M1 phenotype following inflammatory stimuli. It is therefore tempting to speculate that in other myeloid cells such as dendritic cells (DCs), high p16^INK4a^ expression could also influence their inflammatory response under specific environmental conditions.

### Natural killer cell function and cellular senescence

Natural killer (NK) cells are lymphoid cells with immune surveillance functions thanks to their cytotoxic activity and specific cytokine profile. Besides their immune surveillance role against altered cells such as senescent cells within tissues under physiological and pathological conditions, which will be discussed later, recent evidence suggests that senescence onset of NK cell subset can impact their function. Indeed, decidual NK cells have an important role in promoting immune tolerance and maintaining pregnancy (Fu *et al*., 2013). Remarkably, one recent report demonstrates that this specific NK cell subset acquires senescence‐like features including permanent cell cycle arrest, DNA damage accumulation and specific chromatin remodeling. This senescence occurs upon activation of the NK receptor, CD158d, by the soluble HLA‐G, a nonclassical major histocompatibility complex molecule, secreted by fetal trophoblasts. In turn, senescent NK cells produce a specific SASP that positively regulates the neo‐angiogenesis required for embryo implantation during the first trimester of pregnancy (Rajagopalan & Long, 2012; Rajagopalan *et al*., 2014). These findings highlight a positive role of cellular senescence in a physiological immune context and open new perspectives in the NK field. Nevertheless, similar to other immune cells, alterations on NK cells number, phenotype, and function have been reported with aging (Hazeldine & Lord, 2013).

### Cellular senescence alters B‐cell proliferation in aging

The immune response entails waves of cell proliferation followed by extensive cell death and the emergence of memory cells. Thus, the ability of lymphocytes to undergo repeated cell division is essential for effective immune function. Antigen‐specific immune cells have adapted a mechanism otherwise used by malignant cells and germ line cells to extend their replicative capacity, required for lymphocyte function. For instance, in secondary lymphoid organs, naïve B lymphocytes undergo rapid cell division followed by clonal expansion and differentiation from germinal center (GC) B cells to memory B cells (Kelsoe, 1996). Telomere length is significantly increased during the differentiation of naïve into GC B cells (Weng *et al*., 1998). Indeed, quiescent B lymphocytes from peripheral blood samples express low level of telomerase (TERC) (Norrback *et al*., 1996). Following *in vivo* activation and differentiation, TERC levels are transiently induced in GC centroblasts and centrocytes and then down‐regulated again in memory B cells (Hu *et al*., 1997). These results emphasize that TERC re‐expression is possible in somatic B cells in order to maintain their telomere length and prevent senescence. Interestingly, pre‐B lymphocytes isolated from young mouse bone marrow and cultured in the presence of IL‐7 can become senescent (Signer *et al*., 2008). Indeed, after 2 weeks in culture, these cells eventually stop proliferating and concomitantly accumulate p16^INK4a^ and p14/p19^ARF^. It is not known whether B‐cell senescence can naturally occur *in vivo* in young individuals, but with age, the expression levels of both p16^INK4a^ and p14/p19^ARF^ increase in all B lineages, particularly in pro‐B, pre‐B, and IgM+ mature B cells (Krishnamurthy *et al*., 2004; Signer *et al*., 2008). Ectopic expression of p16^INK4a^ or p14/p19^ARF^ in young pro‐/pre‐B cells mimics the effect of aging by decreasing cell growth and survival. In contrast, down‐regulation of the *CDKN2A* locus promotes the proliferative potentials of these cells and *CDKN2A* gene knockout confers upon B cells a predisposition to leukemogenesis, following BCR‐ABL translocation, compared to wild‐type cells. Accordingly, in acute lymphoblastic leukemia, immortalization of B cells induced by BCR‐ABL translocation results in *CDKN2A* locus repression (Williams & Sherr, 2007). Altogether, these findings demonstrate that senescent lymphoid cells accumulate naturally in aging individuals and may prevent B‐cell malignancy.

### T‐cell function, replicative history, and cellular senescence

T lymphocytes are the key mediators of the adaptive immune response. Circulating subpopulations of human T cells have a variety of phenotypes and functions. Briefly, they can be divided into CD4^+^ helper and CD8^+^ cytotoxic T cells. Following the peak of immune cell expansion, most antigen‐specific T cells undergo cell‐mediated apoptosis. The remaining T cells differentiate into long‐lived memory T cells that persist at low frequencies, but retain effector functions and high proliferative potential, allowing them to be on constant surveillance and prevent re‐infection of the host. The most significant age‐related change in the human immune system is the quality and phenotype of the cytotoxic CD8 T‐cell subset. Indeed, with age, and in chronic infections such as human immunodeficiency virus (Appay *et al*., 2007) and cytomegalovirus (Pita‐Lopez *et al*., 2009), the majority of CD8^+^ T cells become antigen‐experienced and acquire a deregulated, pro‐inflammatory phenotype. These late‐differentiated CD8^+^ T cells possess many features of replicative senescence that have been characterized in long‐term *in vitro* cultures (Signer *et al*., 2008). Although CD4^+^ T cells are more resistant to age‐related phenotypic and functional changes than CD8^+^ T cells (Weinberger *et al*., 2007), a progressive increase in the percentage of senescence‐like CD4^+^ T cells is common with increasing age in healthy individuals (Goronzy *et al*., 2007; Czesnikiewicz‐Guzik *et al*., 2008). This increase in senescence‐like CD4^+^ T cells is also observed in patients with chronic infections (Fletcher *et al*., 2005) and autoimmune diseases such as rheumatoid arthritis (RA) (Goronzy *et al*., 2005). Analysis of telomere length in the same healthy individual revealed that naïve CD4^+^ and CD8^+^ T cells have consistently longer telomeres than memory T cells at all ages, suggesting that the differentiation of naïve into memory T cells involves a relatively constant number of cell divisions independent of the donor's age (Weng *et al*., 1998; Effros, 2011). These observations strongly support the notion that telomere length may correlate with the replicative history of T cells during *in vivo* activation and/or differentiation.

Similarly, following multiple rounds of *in vitro* stimulation, T cells progressively undergo a series of cell divisions associated with transient TERC expression that ultimately leads to culture exhaustion exhibiting features of cellular senescence (Effros, 2011). Comparable to other senescent cells, *in vitro* exhausted T cells have short telomeres, cannot proliferate even in the presence of co‐stimulatory molecules, and are resistant to apoptosis and metabolically active. This cell cycle arrest can be overcome by ectopic expression of the catalytic subunit of the telomerase (hTERT), demonstrating a role for telomere erosion in this process (Roth *et al*., 2003). However, exhausted T cells seem to be distinct from senescent T cells because they have low expression of immunological markers of senescence and progressively lose the ability to secrete cytokines in contrast to senescent T cells (Wherry, 2011; Crespo *et al*., 2013). Interestingly, chronic T‐cell activation is not the only approach to obtain senescent T cells. Indeed, T‐cell senescence can also be stress‐induced, the consequence of a deregulated inflammatory environment. For instance, TNF‐α or IFN‐γ, archetypical inflammatory cytokines, can trigger premature senescence of CD8^+^ T cells *in vitro* by activating the stress kinase p38^MAPK^ and down‐regulating hTERT gene expression (Di Mitri *et al*., 2011; Lanna *et al*., 2013). Accordingly, pharmacological inhibition of p38^MAPK^ regulatory pathway such as AMPK–TAB 1 axis delays T‐cell senescence‐induced cell cycle arrest (Lanna *et al*., 2014).

Independent of the nature of the senescence‐inducing signal, all human T cells that enter into senescence and become highly differentiated are characterized by the loss of CD28 expression. CD28 is an essential co‐stimulatory receptor that activates T‐cell proliferation. CD28^null^ T lymphocytes accumulate during aging and cancer presumably due to a prolonged exposure to common persistent antigens (Effros *et al*., 2003; Effros, 2011). They are also found in young individuals as a result of chronic antigenic stimulation (Effros *et al*., 2003; Effros, 2011) or chronic immune degenerative disorders such as juvenile idiopathic arthritis (Dvergsten *et al*., 2013), myelodysplastic syndromes (Xiao *et al*., 2013), idiopathic CD4 lymphopenia (Bignon *et al*., 2015), or RA (Schönland *et al*., 2003). The loss of CD28 is not only a result of T‐cell receptor (TCR) activation. Indeed, in the presence of certain cytokines (IL‐2, IL‐7, and IL‐15), type I interferon (IFN‐α and IFN‐β), and TNF‐α, the CD28 loss is accelerated (for review, see Weng *et al*., 2009). CD28 loss occurs more rapidly among CD8 T cells relative to their CD4 counterparts, presumably due to a faster turnover in the CD8 compartment. CD28^null^ T cells are apoptosis resistant, terminally differentiated effectors with eroded telomeres. This population is, however, very heterogeneous and encompasses both effector, senescent and T‐regulatory (Treg) cells (Freedman *et al*., 1991; Cortesini *et al*., 2001; Chang *et al*., 2002; Suciu‐Foca & Cortesini, 2007).

The mechanism by which CD28 expression is down‐regulated *in vitro* and *in vivo* in senescent T cells has only recently begun to be understood. Mondal *et al*. (2013) reported that alternative dominant‐negative p53 splicing forms, namely Δ133p53 and p53β, are central players during *in vitro* and *in vivo* induced human T‐cell senescence. p53β overexpression or ∆133p53 down‐regulation represses CD28 gene transcription in human cells (Mondal *et al*., 2013). Accordingly, forced expression of ∆133p53 or CD28 in CD8^+^CD28^null^ human T cells is sufficient to delay telomere‐dependent growth arrest following culture exhaustion, but failed to permanently prevent the process (Parish *et al*., 2010).

In addition to low expression of CD28, the absence of CD27, coupled with up‐regulation of CD57 expression in humans (killer cell lectin‐like receptor G1 in mice) and expression of T‐cell immunoglobulin mucin‐3 are thought to be associated with T‐cell senescence (Henson *et al*., 2009). Nevertheless, such alterations of the T‐cell phenotype are not restricted to senescent T cells (for review, see Akbar & Henson, 2011; Wherry, 2011; Crespo *et al*., 2013).

Finally, although senescent T cells are nonresponsive to subsequent stimulation, as are anergic T cells, they are, in contrast, metabolically active and abundantly produce cytokines, including IL‐6 and TNF‐α. Moreover, CD4 senescent T cells also express NK cell‐related receptors and high levels of granzyme B and perforin, which may be important for protection against infections *in vivo* (Appay & Sauce, 2008). Altogether, pro‐inflammatory factors included within the SASP of senescent T cells can cause adverse or positive effects on surrounding nonsenescent cells. For example, human tumor‐induced senescent CD4^+^ and CD8^+^ T‐cell subpopulations are functionally altered because they suppress the proliferation of responder T cells *in vitro*. This suppression requires cell‐to‐cell contact and is considered as a pro‐tumoral mechanism (Montes *et al*., 2008). In contrast, these same tumor‐induced senescent T cells can also modulate monocyte/macrophage cell fate and contribute to antitumoral functions through mechanisms involving galectin 9/Tim3 and CD40/CD40‐L pathways (Ramello *et al*., 2014).

### Hematopoietic progenitors/stem cells and senescence

Cellular senescence not only hallmarks specific differentiated immune cells, as discussed above, but also occurs in hematopoietic progenitors. Hematopoietic stem cells (HSCs) are adult stem cells from which, upon exposure to specific differentiation stimuli, all blood cells originate. In humans and rodent models, the number of HSCs isolated from bone marrow dramatically increases with age, whereas their capacity to proliferate *in vitro* in cloning formation assays (CFU‐F) and to repopulate the bone marrow of irradiated animals progressively decreases (Geiger *et al*., 2013). Several groups have reported that murine HSCs accumulate DNA damage and senescence markers with age (Yahata *et al*., 2011; Flach *et al*., 2014). This is caused not only by an increase in oxidative stress linked to dysfunctions in energy metabolism, but also by the progressive repression of the replication factor MCM4 at each cell division (Flach *et al*., 2014). Defects in HSC replicative capacities could increase the pool of HSCs with impaired differentiation potential over time. Indeed, senescent/aged HSCs show a reduced ability to produce naïve CD4^+^ and CD8^+^ cells and increased differentiation toward the myeloid lineages. Therefore, replicative and age‐induced HSC senescence affects immune system homeostasis (Yahata *et al*., 2011; Flach *et al*., 2014). Accordingly, in mouse models, deletion of two senescence‐associated cell cycle regulators such as p16^INK4A^ and p14/p19^ARF^ increases the *in vitro* HSC replicative potential compared to wild‐type cells (Wang *et al*., 2012). However, because cellular senescence limits tumorigenesis in a cell autonomous‐dependent manner, loss of these two proteins leads to a high prevalence of leukemia (Williams & Sherr, 2007).

Features of senescence are widespread throughout the immune system and result in diverse outcomes. Indeed, one can not only find adverse effects of cellular senescence associated with HSC and lymphocytes in aging and chronic disorders, but also beneficial outcomes such as lineage polarization for M1 macrophages following inflammatory stimuli and the pro‐angiogenic properties for NK cells during pregnancy.

## Cellular senescence and immunity in tissue homeostasis

In response to acute or chronic tissue injury, innate and adaptive immune cells greatly contribute to the maintenance of tissue homeostasis and help ensure the long lifespan of multicellular organisms. Tissue injuries can occur following viral and bacterial infections, or as the consequence of extrinsic or intrinsic organic deficiencies. Under such conditions, injured cells within altered tissues/organs may become functionally deficient leading to either cell death or the onset of cellular senescence. In the latter case, induction of senescence not only prevents the potential proliferation and transformation of damaged/altered cells, but also favors tissue repair through the production of specific factors, called SASP (Tchkonia *et al*., 2013; Demaria *et al*., 2014; Muñoz‐Espín & Serrano, 2014). For instance, senescent fibroblasts and endothelial cells, which transiently accumulate at the wound, start to produce secreted factors important for wound healing. One of them, platelet‐derived growth factor AA, is required for the rapid differentiation of myofibroblasts during wound closure. Accordingly, pharmacological elimination of these senescent cells delayed tissue wound repair in a murine model (Demaria *et al*., 2014).

### Clearance of senescent cells by the immune system

Senescence‐dependent secreted factors also contribute to the recruitment of immune cells participating in cell clearance, particularly of senescent cells following tissue injury. NK cells, macrophages, and cytotoxic CD8^+^ T cells are chemo‐attracted by the inflamed tissues to promote cell death, thus facilitating senescent cell replacement and a return to tissue homeostasis (Hoenicke & Zender, 2012; Davies *et al*., 2013). NK cells are especially important in the immunosurveillance of senescent cells during tissue repair (Krizhanovsky *et al*., 2008a,b). They are attracted to senescent cells through a p53‐dependent secretion of CCL2 [chemokine (C‐C) ligand 2] (Iannello *et al*., 2013). NK cells then recognize these senescent cells through CD58‐ICAM1 binding (Chien *et al*., 2011; Sagiv & Krizhanovsky, 2013). Furthermore, senescent cells express, in an ATM/ATR (ataxia telangiectasia mutated/Rad3‐related kinases)‐dependent manner, ligands for two NK cell activating receptors NKG2D and DNAM1 and secrete IL‐15, a cytokine that promotes NKG2D and DNAM1 expression in NK cells (Krizhanovsky *et al*., 2008a; Soriani *et al*., 2009). Following receptor activation, NK cells can then specifically induce the death of senescent cells through perforin release by exocytosis (Sedelies *et al*., 2008; Sagiv *et al*., 2013). Macrophages also display an immunosurveillance role against senescent cells within tissues under physiological and pathological conditions. For example, during liver fibrosis, p53‐expressing senescent liver satellite cells release IFN‐γ and IL‐6, which in turn skew the polarization of resident Kupffer macrophages and freshly infiltrated macrophages toward the pro‐inflammatory M1 phenotype (Lujambio *et al*., 2013). These M1 macrophages can then rapidly eliminate senescent liver satellite cells within the injured tissue. In contrast, the loss of p53 in senescent liver satellite cells results in IL‐4 production, switching macrophage polarization toward the pro‐survival and pro‐angiogenic M2 phenotype (Lujambio *et al*., 2013).

### Immune cells induce the onset of cellular senescence to maintain tissue homeostasis

Following tissue injury, antigen‐specific CD4^+^ T‐helper 1 (Th1) cells participate in the control of tissue homeostasis in concert with CD8^+^ T cells, macrophages, and NK cells at the inflammatory site. Th1 cells act at two stages during tissue repair. The presence of Th1 cells is required for proper *in vivo* macrophage‐dependent elimination of senescent cells found in damaged tissue, as recently revealed by Kang *et al*. (2011) using a premalignant hepatocyte murine model. Furthermore, Th1 cells can also contribute directly to tissue homeostasis by triggering cellular senescence on tissue‐damaged cells. To reveal this new Th1 cell function, Braumüller *et al*. (2013) used an *in vivo* oncogenic inducible cell transformation system, permitting the expression of one specific cell surface antigen in transformed pancreatic beta cells. Th1 effector cells are antigen‐dependent producers of IFN‐γ and TNF‐α. Once recruited by antigen‐specific expressing beta pancreatic cells, Th1 cells will trigger IFN‐γ‐ and TNF‐α‐induced senescence‐related growth arrest of these interacting beta cancer cells. Senescent beta cancer cells are then rapidly eliminated by the immunosurveillance mechanism that involves NK cells and macrophages (Braumüller *et al*., 2013).

### Inhibiting immune responses through senescence induction

As mentioned, numerous types of immune cells are present at the site of tissue injury to control tissue repair. Recent evidence proposes that induction of cellular senescence is also a mechanism used by the immune system, through the action of regulatory T (Tregs) cells, to restrain immune responses.

Treg cells are regulators of the adaptive immune response. They are therefore crucial for the maintenance of immune self‐tolerance and homeostasis (for review, see Sakaguchi *et al*., 2008). The most studied Treg cells are the CD4^+^CD25^hi^FoxP3^+^ that are required for self‐tolerance and a proper immune response to pathogens (Sakaguchi, 2005). Recently, Ye *et al*. (2012) demonstrated that CD4^+^CD25^hi^FoxP3^+^ Treg cells impaired T‐cell proliferation by inducing senescence in effector T cells, therefore increasing the methods used by Treg to display a suppressive activity (for review, see Sojka *et al*., 2008; Wing & Sakaguchi, 2012). Actually, CD4^+^CD25^hi^FoxP3^+^ Tregs promote T‐cell growth arrest through activation of the p38^MAPK^ and p53 signaling pathways that control two cell cycle inhibitors p16^INK4a^ and p21^WAF1^, respectively (Ye *et al*., 2012). The resulting senescent T cells harbor a specific secretome characterized by IL‐6/IL‐8/IL‐10/TGF‐β/IFN‐γ/TNF‐α production, down‐regulation of surface markers, such as CD28 and CD27, and up‐regulation of PD‐1. This senescence induction by CD4^+^CD25^hi^FoxP3^+^ can be prevented through the activation of the TLR8 receptors (Ye *et al*., 2012). Interestingly, these induced senescent T cells become themselves regulatory and inhibit the proliferation of responding CD4 T cells. Another recent study showed that the tumor suppressor gene p53 participates in the *in vivo* generation of FoxP3 Treg cells from naive CD4^+^ cells (Kawashima *et al*., 2013). Indeed, p53 protein levels increase in CD4^+^ T cells following TCR activation and several p53 binding sites are present on the FoxP3 promoter. As expected, specific inactivation of p53 in CD4^+^ T cells results in a dramatic reduction in CD4^+^CD25^hi^FoxP3 Tregs in mouse models (Kawashima *et al*., 2013). These findings reveal the complex interplay between senescence inducers, such as p53, and immune cell fate.

Finally, recent data from Burzyn *et al*. (2013) revealed a surprising and novel function for Tregs. Following intramuscular injection of cardiotoxin as a model of induced tissue damage following acute injury in mice, the authors unveiled the important role of Tregs in muscle tissue protection, repair, and maintenance. This mode of action seems to be independent of their immunosuppressive functions (Arpaia *et al*., 2015). Interestingly, transient accumulation of senescent myogenic cells is also required for proper muscle repair and participates in the process of skeletal muscle regeneration as well (Le Roux *et al*., 2015). Investigating the link between Tregs, cellular senescence, and tissue homeostasis could provide new avenues of research in the field.

## Summary and perspectives: an integrated view of cellular senescence as a homeostatic orchestrator in immune cell fate and function

In conclusion, we propose in this review an integrated view of cellular senescence features in the immune system and its homeostatic roles throughout life. We described how intrinsic senescence‐inducing actors could be part of the terminal differentiation program of several immune cell types, such as megakaryocytes, T cells, macrophages, and probably DCs. Markers of senescence, including *CDKN2A*, are also involved in the decision by monocytes to differentiate toward inflammatory M1 macrophages, which in turn will participate in T‐lymphocyte polarization toward senescence‐inducing Th1 cells. CD4^+^CD25^Hi^FoxP3^+^ Treg cells exercise their immunosuppressive functions by also inducing senescence on inflammatory T cells, which in turn will be converted into new immunomodulatory cells. Although senescence can be eventually propagated from cell to cell by a bystander effect (Acosta *et al*., 2013), the mechanism underlying the suppressive effect of senescent T cells remains largely unknown.

Following acute or chronic tissue injuries, cellular senescence in the immune system is central for tissue homeostasis. Specific immune cells, via cell autonomous and non‐cell autonomous mechanisms, use senescence‐induced cell cycle arrest and secretory properties to control the outcome of tissue development and tissue repair throughout ontogeny. Furthermore, depending on the cell type affected by senescence and the microenvironment, where tissue‐resident senescent cells accumulate, the senescence‐inducing secretome allows the attraction of the most specific and relevant immune cells required for their own elimination. This secretome influences not only the types and quantity of attracted immune cells, but also their cell fate decision, as exemplified by M1 macrophages and T‐cell‐induced senescence in preneoplastic lesions (Burd *et al*., 2013). Specifically targeting this senescence‐inducing secretome might represent a therapeutic alternative against chronic degenerative diseases. Thus, anti‐TNF‐α therapy in patients with RA was used to restore telomerase expression in T lymphocytes, thus delaying RA‐induced premature T senescence (Bryl *et al*., 2005).

With aging, the immune system suffers from both impaired HSC self‐renewal and a shift of HSC pluripotency toward myeloid lineages. Furthermore, reduction in functional lymphoid‐derived cells altogether with an increase in the number of immune and tissue‐senescent cells, which cannot be properly eliminated, will also progressively contribute to the age‐dependent loss of tissue function. These discoveries open new perspectives for innovative therapies to delay/restore functional organs in the elderly or in patients with chronic immune degenerative disorders.

## Funding

Work leading to this review was supported in part by research funding from IMTO‐Vieillissement‐Fond d'Amorçage to BJM and by institutional fundings from Inserm and University to CJ. RV is a recipient of a postdoctoral fellowship from European Union project Innovative Medicine Initiative (‘BeTheCure’; contract number 115142–2 to C.J.).

## Conflict of interest

The authors declare no competing financial interests.

## References

[acel12455-bib-0001] Acosta JC , Banito A , Wuestefeld T , Georgilis A , Janich P , Morton JP , Athineos D , Kang TW , Lasitschka F , Andrulis M , Pascual G , Morris KJ , Khan S , Jin H , Dharmalingam G , Snijders AP , Carroll T , Capper D , Pritchard C , Inman GJ , Longerich T , Sansom OJ , Benitah SA , Zender L , Gil J (2013) A complex secretory program orchestrated by the inflammasome controls paracrine senescence. Nat. Cell Biol. 15, 978–990. 23770676 10.1038/ncb2784 PMC3732483

[acel12455-bib-0002] Akbar AN , Henson SM (2011) Are senescence and exhaustion intertwined or unrelated processes that compromise immunity? Nat. Rev. Immunol. 11, 289–295. 21436838 10.1038/nri2959

[acel12455-bib-0003] Appay V , Sauce D (2008) Immune activation and inflammation in HIV‐1 infection: causes and consequences. J. Pathol. 214, 231–241. 18161758 10.1002/path.2276

[acel12455-bib-0004] Appay V , Almeida JR , Sauce D , Autran B , Papagno L (2007) Accelerated immune senescence and HIV‐1 infection. Exp. Gerontol. 42, 432–437. 17307327 10.1016/j.exger.2006.12.003

[acel12455-bib-0005] Arpaia N , Green JA , Moltedo B , Arvey A , Hemmers S , Yuan S , Treuting PM , Rudensky AY (2015) A distinct function of regulatory T cells in tissue protection. Cell 162, 1078–1089. 26317471 10.1016/j.cell.2015.08.021 PMC4603556

[acel12455-bib-0006] Bachoo RM , Maher EA , Ligon KL , Sharpless NE , Chan SS , You MJ , Tang Y , DeFrances J , Stover E , Weissleder R , Rowitch DH , Louis DN , DePinho RA (2002) Epidermal growth factor receptor and Ink4a/Arf: convergent mechanisms governing terminal differentiation and transformation along the neural stem cell to astrocyte axis. Cancer Cell 1, 269–277. 12086863 10.1016/s1535-6108(02)00046-6

[acel12455-bib-0007] Besancenot R , Chaligné R , Tonetti C , Pasquier F , Marty C , Lécluse Y , Vainchenker W , Constantinescu SN , Giraudier S (2010) A senescence‐like cell‐cycle arrest occurs during megakaryocytic maturation: implications for physiological and pathological megakaryocytic proliferation. PLoS Biol. 8, pii: e1000476. 20838657 10.1371/journal.pbio.1000476 PMC2935456

[acel12455-bib-0008] Bignon A , Régent A , Klipfel L , Desnoyer A , de la Grange P , Martinez V , Lortholary O , Dalloul A , Mouthon L , Balabanian K (2015) DUSP4‐mediated accelerated T‐cell senescence in idiopathic CD4 lymphopenia. Blood 125, 2507–2518. 25733583 10.1182/blood-2014-08-598565

[acel12455-bib-0009] Biswas SK , Mantovani A (2010) Macrophage plasticity and interaction with lymphocyte subsets: cancer as a paradigm. Nat. Immunol. 11, 889–896. 20856220 10.1038/ni.1937

[acel12455-bib-0010] Braumüller H , Wieder T , Brenner E , Aßmann S , Hahn M , Alkhaled M , Schilbach K , Essmann F , Kneilling M , Griessinger C , Ranta F , Ullrich S , Mocikat R , Braungart K , Mehra T , Fehrenbacher B , Berdel J , Niessner H , Meier F , van den Broek M , Häring HU , Handgretinger R , Quintanilla‐Martinez L , Fend F , Pesic M , Bauer J , Zender L , Schaller M , Schulze‐Osthoff K , Röcken M (2013) T‐helper‐1‐cell cytokines drive(9) cancer into senescence. Nature 494, 361–365. 23376950 10.1038/nature11824

[acel12455-bib-0011] Bryl E , Vallejo AN , Matteson EL , Witkowski JM , Weyand CM , Goronzy JJ (2005) Modulation of CD28 expression with anti‐tumor necrosis factor alpha therapy in rheumatoid arthritis. Arthritis Rheum. 52, 2996–3003. 16200579 10.1002/art.21353

[acel12455-bib-0012] Burd CE , Sorrentino JA , Clark KS , Darr DB , Krishnamurthy J , Deal AM , Bardeesy N , Castrillon DH , Beach DH , Sharpless NE (2013) Monitoring tumorigenesis and senescence *in vivo* with a p16(INK4a)‐luciferase model. Cell 152, 340–351. 23332765 10.1016/j.cell.2012.12.010 PMC3718011

[acel12455-bib-0013] Burzyn D , Kuswanto W , Kolodin D , Shadrach JL , Cerletti M , Jang Y , Sefik E , Tan TG , Wagers AJ , Benoist C , Mathis D (2013) A special population of regulatory T cells potentiates muscle repair. Cell 155, 1282–1295. 24315098 10.1016/j.cell.2013.10.054 PMC3894749

[acel12455-bib-0014] Campisi J , Robert L (2014) Cell senescence: role in aging and age‐related diseases. Interdiscip. Top. Gerontol. 39, 45–61. 24862014 10.1159/000358899 PMC4211612

[acel12455-bib-0015] Chang CC , Ciubotariu R , Manavalan JS , Yuan J , Colovai AI , Piazza F , Lederman S , Colonna M , Cortesini R , Dalla‐Favera R , Suciu‐Foca N (2002) Tolerization of dendritic cells by T(S) cells: the crucial role of inhibitory receptors ILT3 and ILT4. Nat. Immunol. 3, 237–243. 11875462 10.1038/ni760

[acel12455-bib-0016] Chien Y , Scuoppo C , Wang X , Fang X , Balgley B , Bolden JE , Premsrirut P , Luo W , Chicas A , Lee CS , Kogan SC , Lowe SW (2011) Control of the senescence‐associated secretory phenotype by NF‐{kappa}B promotes senescence and enhances chemosensitivity. Genes Dev. 25, 2125–2136. 21979375 10.1101/gad.17276711 PMC3205583

[acel12455-bib-0017] Chou JP , Effros RB (2013) T cell replicative senescence in human aging. Curr. Pharm. Des. 19, 1680–1698. 23061726 10.2174/138161213805219711 PMC3749774

[acel12455-bib-0018] Cortesini R , LeMaoult J , Ciubotariu R , Cortesini NS (2001) CD8+CD28‐ T suppressor cells and the induction of antigen‐specific, antigen‐presenting cell‐mediated suppression of Th reactivity. Immunol. Rev. 182, 201–206. 11722635 10.1034/j.1600-065x.2001.1820116.x

[acel12455-bib-0019] Crespo J , Sun H , Welling TH , Tian Z , Zou W (2013) T cell anergy, exhaustion, senescence, and stemness in the tumor microenvironment. Curr. Opin. Immunol. 25, 214–221. 23298609 10.1016/j.coi.2012.12.003 PMC3636159

[acel12455-bib-0020] Cudejko C , Wouters K , Fuentes L , Hannou SA , Paquet C , Bantubungi K , Bouchaert E , Vanhoutte J , Fleury S , Remy P , Tailleux A , Chinetti‐Gbaguidi G , Dombrowicz D , Staels B , Paumelle R (2011) p16INK4a deficiency promotes IL‐4‐induced polarization and inhibits proinflammatory signaling in macrophages. Blood 118, 2556–2566. 21636855 10.1182/blood-2010-10-313106 PMC3677739

[acel12455-bib-0021] Czesnikiewicz‐Guzik M , Lee WW , Cui D , Hiruma Y , Lamar DL , Yang ZZ , Ouslander JG , Weyand CM , Goronzy JJ (2008) T cell subset‐specific susceptibility to aging. Clin. Immunol. 127, 107–118. 18222733 10.1016/j.clim.2007.12.002 PMC2435295

[acel12455-bib-0022] Davies LC , Jenkins SJ , Allen JE , Taylor PR (2013) Tissue‐resident macrophages. Nat. Immunol. 14, 986–995. 24048120 10.1038/ni.2705 PMC4045180

[acel12455-bib-0023] Demaria M , Ohtani N , Youssef SA , Rodier F , Toussaint W , Mitchell JR , Laberge RM , Vijg J , Van Steeg H , Dollé ME , Hoeijmakers JH , de Bruin A , Hara E , Campisi J (2014) An essential role for senescent cells in optimal wound healing through secretion of PDGF‐AA. Dev. Cell 31, 722–733. 25499914 10.1016/j.devcel.2014.11.012 PMC4349629

[acel12455-bib-0024] Di Mitri D , Azevedo RI , Henson SM , Libri V , Riddell NE , Macaulay R , Kipling D , Soares MV , Battistini L , Akbar AN (2011) Reversible senescence in human CD4+CD45RA+CD27‐ memory T cells. J. Immunol. 187, 2093–2100. 21788446 10.4049/jimmunol.1100978

[acel12455-bib-0025] Dvergsten JA , Mueller RG , Griffin P , Abedin S , Pishko A , Michel JJ , Rosenkranz ME , Reed AM , Kietz DA , Vallejo AN (2013) Premature cell senescence and T cell receptor‐independent activation of CD8+ T cells in juvenile idiopathic arthritis. Arthritis Rheum. 65, 2201–2210. 23686519 10.1002/art.38015 PMC3729743

[acel12455-bib-0026] Effros RB (2011) Telomere/telomerase dynamics within the human immune system: effect of chronic infection and stress. Exp. Gerontol. 46, 135–140. 20833238 10.1016/j.exger.2010.08.027 PMC3246363

[acel12455-bib-0027] Effros RB , Dagarag M , Valenzuela HF (2003) *In vitro* senescence of immune cells. Exp. Gerontol. 38, 1243–1249. 14698803 10.1016/j.exger.2003.09.004

[acel12455-bib-0028] Flach J , Bakker ST , Mohrin M , Conroy PC , Pietras EM , Reynaud D , Alvarez S , Diolaiti ME , Ugarte F , Forsberg EC , Le Beau MM , Stohr BA , Méndez J , Morrison CG , Passegué E (2014) Replication stress is a potent driver of functional decline in ageing haematopoietic stem cells. Nature 512, 198–202. 25079315 10.1038/nature13619 PMC4456040

[acel12455-bib-0029] Fletcher JM , Vukmanovic‐Stejic M , Dunne PJ , Birch KE , Cook JE , Jackson SE , Salmon M , Rustin MH , Akbar AN (2005) Cytomegalovirus‐specific CD4+ T cells in healthy carriers are continuously driven to replicative exhaustion. J. Immunol. 175, 8218–8225. 16339561 10.4049/jimmunol.175.12.8218

[acel12455-bib-0030] Freedman MS , Ruijs TC , Blain M , Antel JP (1991) Phenotypic and functional characteristics of activated CD8+ cells: a CD11b‐CD28‐ subset mediates noncytolytic functional suppression. Clin. Immunol. Immunopathol. 60, 254–267. 1649028 10.1016/0090-1229(91)90068-l

[acel12455-bib-0031] Fu B , Li X , Sun R , Tong X , Ling B , Tian Z , Wei H (2013) Natural killer cells promote immune tolerance by regulating inflammatory TH17 cells at the human maternal‐fetal interface. Proc. Natl Acad. Sci. USA 110, E231–E240. 23271808 10.1073/pnas.1206322110 PMC3549088

[acel12455-bib-0032] Fuentes L , Wouters K , Hannou SA , Cudejko C , Rigamonti E , Mayi TH , Derudas B , Pattou F , Chinetti‐Gbaguidi G , Staels B , Paumelle R (2011) Downregulation of the tumour suppressor p16INK4A contributes to the polarisation of human macrophages toward an adipose tissue macrophage (ATM)‐like phenotype. Diabetologia 54, 3150–3156. 21968977 10.1007/s00125-011-2324-0 PMC4020795

[acel12455-bib-0033] Geiger H , de Haan G , Florian MC (2013) The ageing haematopoietic stem cell compartment. Nat. Rev. Immunol. 13, 376–389. 23584423 10.1038/nri3433

[acel12455-bib-0034] Gil J , Peters G (2006) Regulation of the INK4b‐ARF‐INK4a tumour suppressor locus: all for one or one for all. Nat. Rev. Mol. Cell Biol. 7, 667–677. 16921403 10.1038/nrm1987

[acel12455-bib-0035] Gordon S , Martinez FO (2010) Alternative activation of macrophages: mechanism and functions. Immunity 32, 593–604. 20510870 10.1016/j.immuni.2010.05.007

[acel12455-bib-0036] Gordon S , Taylor PR (2005) Monocyte and macrophage heterogeneity. Nat. Rev. Immunol. 5, 953–964. 16322748 10.1038/nri1733

[acel12455-bib-0037] Goronzy JJ , Henel G , Sawai H , Singh K , Lee EB , Pryshchep S , Weyand CM (2005) Costimulatory pathways in rheumatoid synovitis and T‐cell senescence. Ann. N. Y. Acad. Sci. 1062, 182–194. 16461801 10.1196/annals.1358.022

[acel12455-bib-0038] Goronzy JJ , Lee WW , Weyand CM (2007) Aging and T‐cell diversity. Exp. Gerontol. 42, 400–406. 17218073 10.1016/j.exger.2006.11.016 PMC2680153

[acel12455-bib-0039] Hayflick L , Moorhead PS (1961) The serial cultivation of human diploid cell strains. Exp. Cell Res. 25, 585–621. 13905658 10.1016/0014-4827(61)90192-6

[acel12455-bib-0040] Hazeldine J , Lord JM (2013) The impact of ageing on natural killer cell function and potential consequences for health in older adults. Ageing Res. Rev. 12, 1069–1078. 23660515 10.1016/j.arr.2013.04.003 PMC4147963

[acel12455-bib-0041] Henson SM , Franzese O , Macaulay R , Libri V , Azevedo RI , Kiani‐Alikhan S , Plunkett FJ , Masters JE , Jackson S , Griffiths SJ , Pircher HP , Soares MV , Akbar AN (2009) KLRG1 signaling induces defective Akt (ser473) phosphorylation and proliferative dysfunction of highly differentiated CD8+ T cells. Blood 113, 6619–6628. 19406987 10.1182/blood-2009-01-199588

[acel12455-bib-0042] Hoenicke L , Zender L (2012) Immune surveillance of senescent cells – biological significance in cancer‐ and non‐cancer pathologies. Carcinogenesis 33, 1123–1126. 22470164 10.1093/carcin/bgs124

[acel12455-bib-0043] Hu BT , Lee SC , Marin E , Ryan DH , Insel RA (1997) Telomerase is up‐regulated in human germinal center B cells *in vivo* and can be re‐expressed in memory B cells activated *in vitro* . J. Immunol. 159, 1068–1071. 9233598

[acel12455-bib-0044] Iannello A , Thompson TW , Ardolino M , Lowe SW , Raulet DH (2013) p53‐dependent chemokine production by senescent tumor cells supports NKG2D‐dependent tumor elimination by natural killer cells. J. Exp. Med. 210, 2057–2069. 24043758 10.1084/jem.20130783 PMC3782044

[acel12455-bib-0045] Kang TW , Yevsa T , Woller N , Hoenicke L , Wuestefeld T , Dauch D , Hohmeyer A , Gereke M , Rudalska R , Potapova A , Iken M , Vucur M , Weiss S , Heikenwalder M , Khan S , Gil J , Bruder D , Manns M , Schirmacher P , Tacke F , Ott M , Luedde T , Longerich T , Kubicka S , Zender L (2011) Senescence surveillance of pre‐malignant hepatocytes limits liver cancer development. Nature 479, 547–551. 22080947 10.1038/nature10599

[acel12455-bib-0046] Kawashima H , Takatori H , Suzuki K , Iwata A , Yokota M , Suto A , Minamino T , Hirose K , Nakajima H (2013) Tumor suppressor p53 inhibits systemic autoimmune diseases by inducing regulatory T cells. J. Immunol. 191, 3614–3623. 24006461 10.4049/jimmunol.1300509

[acel12455-bib-0047] Kelsoe G (1996) Life and death in germinal centers (redux). Immunity 4, 107–111. 8624801 10.1016/s1074-7613(00)80675-5

[acel12455-bib-0048] Krishnamurthy J , Torrice C , Ramsey MR , Kovalev GI , Al‐Regaiey K , Su L , Sharpless NE (2004) Ink4a/Arf expression is a biomarker of aging. J. Clin. Invest. 114, 1299–1307. 15520862 10.1172/JCI22475 PMC524230

[acel12455-bib-0049] Krizhanovsky V , Xue W , Zender L , Yon M , Hernando E , Lowe SW (2008a) Implications of cellular senescence in tissue damage response, tumor suppression, and stem cell biology. Cold Spring Harb. Symp. Quant. Biol. 73, 513–522. 19150958 10.1101/sqb.2008.73.048 PMC3285266

[acel12455-bib-0050] Krizhanovsky V , Yon M , Dickins RA , Hearn S , Simon J , Miething C , Yee H , Zender L , Lowe SW (2008b) Senescence of activated stellate cells limits liver fibrosis. Cell 134, 657–667. 18724938 10.1016/j.cell.2008.06.049 PMC3073300

[acel12455-bib-0051] Lanna A , Coutavas E , Levati L , Seidel J , Rustin MH , Henson SM , Akbar AN , Franzese O (2013) IFN‐α inhibits telomerase in human CD8⁺ T cells by both hTERT downregulation and induction of p38 MAPK signaling. J. Immunol. 191, 3744–3752. 23997212 10.4049/jimmunol.1301409 PMC3836248

[acel12455-bib-0052] Lanna A , Henson SM , Escors D , Akbar AN (2014) The kinase p38 activated by the metabolic regulator AMPK and scaffold TAB 1 drives the senescence of human T cells. Nat. Immunol. 15, 965–972. 25151490 10.1038/ni.2981 PMC4190666

[acel12455-bib-0053] Le Roux I , Konge J , Le Cam L , Flamant P , Tajbakhsh S (2015) Numb is required to prevent p53‐dependent senescence following skeletal muscle injury. Nat. Commun. 6, 8528. 26503169 10.1038/ncomms9528 PMC4639798

[acel12455-bib-0054] Lujambio A , Akkari L , Simon J , Grace D , Tschaharganeh DF , Bolden JE , Zhao Z , Thapar V , Joyce JA , Krizhanovsky V , Lowe SW (2013) Non‐cell‐autonomous tumor suppression by p53. Cell 153, 449–460. 23562644 10.1016/j.cell.2013.03.020 PMC3702034

[acel12455-bib-0055] Mantovani A , Biswas SK , Galdiero MR , Sica A , Locati M (2013) Macrophage plasticity and polarization in tissue repair and remodelling. J. Pathol. 229, 176–185. 23096265 10.1002/path.4133

[acel12455-bib-0056] Mondal AM , Horikawa I , Pine SR , Fujita K , Morgan KM , Vera E , Mazur SJ , Appella E , Vojtesek B , Blasco MA , Lane DP , Harris CC (2013) p53 isoforms regulate aging‐ and tumor‐associated replicative senescence in T lymphocytes. J. Clin. Invest. 123, 5247–5257. 24231352 10.1172/JCI70355 PMC3859419

[acel12455-bib-0057] Montes CL , Chapoval AI , Nelson J , Orhue V , Zhang X , Schulze DH , Strome SE , Gastman BR (2008) Tumor‐induced senescent T cells with suppressor function: a potential form of tumor immune evasion. Cancer Res. 68, 870–879. 18245489 10.1158/0008-5472.CAN-07-2282

[acel12455-bib-0058] Mosser DM , Edwards JP (2008) Exploring the full spectrum of macrophage activation. Nat. Rev. Immunol. 8(12), 958–969. 19029990 10.1038/nri2448 PMC2724991

[acel12455-bib-0059] Muñoz‐Espín D , Serrano M (2014) Cellular senescence: from physiology to pathology. Nat. Rev. Mol. Cell Biol. 15, 482–496. 24954210 10.1038/nrm3823

[acel12455-bib-0060] Murakami Y , Mizoguchi F , Saito T , Miyasaka N , Kohsaka H (2012) p16(INK4a) exerts an anti‐inflammatory effect through accelerated IRAK1 degradation in macrophages. J. Immunol. 189, 5066–5072. 23066149 10.4049/jimmunol.1103156

[acel12455-bib-0061] Norrback KF , Dahlenborg K , Carlsson R , Roos G (1996) Telomerase activation in normal B lymphocytes and non‐Hodgkin's lymphomas. Blood 88, 222–229. 8704177

[acel12455-bib-0062] Pajcini KV , Corbel SY , Sage J , Pomerantz JH , Blau HM (2010) Transient inactivation of Rb and ARF yields regenerative cells from postmitotic mammalian muscle. Cell Stem Cell 7, 198–213. 20682446 10.1016/j.stem.2010.05.022 PMC2919350

[acel12455-bib-0063] Paramio JM , Segrelles C , Ruiz S , Martin‐Caballero J , Page A , Martinez J , Serrano M , Jorcano JL (2001) The ink4a/arf tumor suppressors cooperate with p21cip1/waf in the processes of mouse epidermal differentiation, senescence, and carcinogenesis. J. Biol. Chem. 276, 44203–44211. 11551927 10.1074/jbc.M105650200

[acel12455-bib-0064] Parish ST , Wu JE , Effros RB (2010) Sustained CD28 expression delays multiple features of replicative senescence in human CD8 T lymphocytes. J. Clin. Immunol. 30, 798–805. 20721608 10.1007/s10875-010-9449-7 PMC2970803

[acel12455-bib-0065] Philipot D , Guérit D , Platano D , Chuchana P , Olivotto E , Espinoza F , Dorandeu A , Pers YM , Piette J , Borzi RM , Jorgensen C , Noel D , Brondello JM (2014) p16INK4a and its regulator miR‐24 link senescence and chondrocyte terminal differentiation‐associated matrix remodelling in osteoarthritis. Arthritis. Res. Ther. 16, R58. 24572376 10.1186/ar4494 PMC4060445

[acel12455-bib-0066] Pita‐Lopez ML , Gayoso I , DelaRosa O , Casado JG , Alonso C , Muñoz‐Gomariz E , Tarazona R , Solana R (2009) Effect of ageing on CMV‐specific CD8 T cells from CMV seropositive healthy donors. Immun. Ageing 6, 11. 19715573 10.1186/1742-4933-6-11 PMC2741428

[acel12455-bib-0067] Rajagopalan S , Long EO (2012) Cellular senescence induced by CD158d reprograms natural killer cells to promote vascular remodeling. Proc. Natl Acad. Sci. USA 109, 20596–20601. 23184984 10.1073/pnas.1208248109 PMC3528503

[acel12455-bib-0068] Rajagopalan S , Lee EC , DuPrie ML , Long EO (2014) TNFR‐associated factor 6 and TGF‐β‐activated kinase 1 control signals for a senescence response by an endosomal NK cell receptor. J. Immunol. 192, 714–721. 24337384 10.4049/jimmunol.1302384 PMC4556140

[acel12455-bib-0069] Ramello MC , Boari JT , Canale FP , Mena HA , Negrotto S , Gastman B , Gruppi A , Rodríguez EV , Montes CL (2014) Tumor‐induced senescent T cells promote the secretion of pro‐inflammatory cytokines and angiogenic factors by human monocytes/macrophages through a mechanism that involves Tim‐3 and CD40L. Cell Death Dis. 5, e1507. 25375372 10.1038/cddis.2014.451 PMC4260722

[acel12455-bib-0070] Roth A , Yssel H , Pene J , Chavez EA , Schertzer M , Lansdorp PM , Spits H , Luiten RM (2003) Telomerase levels control the lifespan of human T lymphocytes. Blood 102, 849–857. 12689947 10.1182/blood-2002-07-2015

[acel12455-bib-0071] Sagiv A , Krizhanovsky V (2013) Immunosurveillance of senescent cells: the bright side of the senescence program. Biogerontology 14, 617–628. 24114507 10.1007/s10522-013-9473-0

[acel12455-bib-0072] Sagiv A , Biran A , Yon M , Simon J , Lowe SW , Krizhanovsky V (2013) Granule exocytosis mediates immune surveillance of senescent cells. Oncogene 32, 1971–1977. 22751116 10.1038/onc.2012.206 PMC3630483

[acel12455-bib-0073] Sakaguchi S (2005) Naturally arising Foxp3‐expressing CD25+CD4+ regulatory T cells in immunological tolerance to self and non‐self. Nat. Immunol. 6, 345–352. 15785760 10.1038/ni1178

[acel12455-bib-0074] Sakaguchi S , Yamaguchi T , Nomura T , Ono M (2008) Regulatory T cells and immune tolerance. Cell 133, 775–787. 18510923 10.1016/j.cell.2008.05.009

[acel12455-bib-0075] Salama R , Sadaie M , Hoare M , Narita M (2014) Cellular senescence and its effector programs. Genes Dev. 28, 99–114. 24449267 10.1101/gad.235184.113 PMC3909793

[acel12455-bib-0076] Schönland SO , Lopez C , Widmann T , Zimmer J , Bryl E , Goronzy JJ , Weyand CM (2003) Premature telomeric loss in rheumatoid arthritis is genetically determined and involves both myeloid and lymphoid cell lineages. Proc. Natl Acad. Sci. USA 100, 13471–13476. 14578453 10.1073/pnas.2233561100 PMC263838

[acel12455-bib-0077] Sedelies KA , Ciccone A , Clarke CJ , Oliaro J , Sutton VR , Scott FL , Silke J , Susanto O , Green DR , Johnstone RW , Bird PI , Trapani JA , Waterhouse NJ (2008) Blocking granule‐mediated death by primary human NK cells requires both protection of mitochondria and inhibition of caspase activity. Cell Death Differ. 15, 708–717. 18202705 10.1038/sj.cdd.4402300

[acel12455-bib-0078] Signer RA , Montecino‐Rodriguez E , Witte ON , Dorshkind K (2008) Aging and cancer resistance in lymphoid progenitors are linked processes conferred by p16Ink4a and Arf. Genes Dev. 22, 3115–3120. 19056891 10.1101/gad.1715808 PMC2593609

[acel12455-bib-0079] Sojka DK , Huang YH , Fowell DJ (2008) Mechanisms of regulatory T‐cell suppression – a diverse arsenal for a moving target. Immunology 124, 13–22. 18346152 10.1111/j.1365-2567.2008.02813.x PMC2434375

[acel12455-bib-0080] Soriani A , Zingoni A , Cerboni C , Iannitto ML , Ricciardi MR , Di Gialleonardo V , Cippitelli M , Fionda C , Petrucci MT , Guarini A , Foà R , Santoni A (2009) ATM‐ATR‐dependent up‐regulation of DNAM‐1 and NKG2D ligands on multiple myeloma cells by therapeutic agents results in enhanced NK‐cell susceptibility and is associated with a senescent phenotype. Blood 113, 3503–3511. 19098271 10.1182/blood-2008-08-173914

[acel12455-bib-0081] Suciu‐Foca N , Cortesini R (2007) Central role of ILT3 in the T suppressor cell cascade. Cell. Immunol. 248, 59–67. 17923119 10.1016/j.cellimm.2007.01.013

[acel12455-bib-0082] Tchkonia T , Zhu Y , van Deursen J , Campisi J , Kirkland JL (2013) Cellular senescence and the senescent secretory phenotype: therapeutic opportunities. J. Clin. Invest. 123, 966–972. 23454759 10.1172/JCI64098 PMC3582125

[acel12455-bib-0083] Wang J , Sun Q , Morita Y , Jiang H , Gross A , Lechel A , Hildner K , Guachalla L , Gompf A , Hartmann D , Schambach A , Wuestefeld T , Dauch D , Schrezenmeier H , Hofmann W‐K , Nakauchi H , Ju Z , Kestler H , Zender L , Rudolph K (2012) A differentiation checkpoint limits hematopoietic stem cell self‐renewal in response to DNA damage. Cell 148, 1001–1015. 22385964 10.1016/j.cell.2012.01.040

[acel12455-bib-0084] Weinberger B , Lazuardi L , Weiskirchner I , Keller M , Neuner C , Fischer KH , Neuman B , Würzner R , Grubeck‐Loebenstein B (2007) Healthy aging and latent infection with CMV lead to distinct changes in CD8+ and CD4+ T‐cell subsets in the elderly. Hum. Immunol. 68, 86–90. 17321897 10.1016/j.humimm.2006.10.019

[acel12455-bib-0085] Weng NP , Hathcock KS , Hodes RJ (1998) Regulation of telomere length and telomerase in T and B cells: a mechanism for maintaining replicative potential. Immunity 9, 151–157. 9729035 10.1016/s1074-7613(00)80597-x

[acel12455-bib-0086] Weng NP , Akbar AN , Goronzy J (2009) CD28(‐) T cells: their role in the age‐associated decline of immune function. Trends Immunol. 30, 306–312. 19540809 10.1016/j.it.2009.03.013 PMC2801888

[acel12455-bib-0087] Wherry EJ (2011) T cell exhaustion. Nat. Immunol. 12, 492–499. 21739672 10.1038/ni.2035

[acel12455-bib-0088] Williams RT , Sherr CJ (2007) The ARF tumor suppressor in acute leukemias: insights from mouse models of Bcr‐Abl‐induced acute lymphoblastic leukemia. Adv. Exp. Med. Biol. 604, 107–114. 17695724 10.1007/978-0-387-69116-9_9

[acel12455-bib-0089] Wing JB , Sakaguchi S (2012) Multiple treg suppressive modules and their adaptability. Front Immunol. 3, 178. 22754556 10.3389/fimmu.2012.00178 PMC3386489

[acel12455-bib-0090] Wynn TA , Chawla A , Pollard JW (2013) Macrophage biology in development, homeostasis and disease. Nature 496, 445–455. 23619691 10.1038/nature12034 PMC3725458

[acel12455-bib-0091] Xiao Y , Wang J , Song H , Zou P , Zhou D , Liu L (2013) CD34+ cells from patients with myelodysplastic syndrome present different p21 dependent premature senescence. Leuk. Res. 37, 333–340. 23219618 10.1016/j.leukres.2012.11.006

[acel12455-bib-0092] Yahata T , Takanashi T , Muguruma Y , Ibrahim AA , Matsuzawa H , Uno T , Sheng Y , Onizuka M , Ito M , Kato S , Ando K (2011) Accumulation of oxidative DNA damage restricts the self‐renewal capacity of human hematopoietic stem cells. Blood 118, 2941–2950. 21734240 10.1182/blood-2011-01-330050

[acel12455-bib-0093] Ye J , Huang X , Hsueh EC , Zhang Q , Ma C , Zhang Y , Varvares MA , Hoft DF , Peng G (2012) Human regulatory T cells induce T‐lymphocyte senescence. Blood 120, 2021–2031. 22723548 10.1182/blood-2012-03-416040 PMC3437594

